# Genome Sequence Analysis of the Biogenic Amine-Producing Strain *Lactococcus lactis* subsp. *cremoris* CECT 8666 (Formerly GE2-14)

**DOI:** 10.1128/genomeA.01088-14

**Published:** 2014-10-23

**Authors:** Victor Ladero, Beatriz del Rio, Daniel M. Linares, Maria Fernandez, Baltasar Mayo, M. Cruz Martin, Miguel A. Alvarez

**Affiliations:** Instituto de Productos Lácteos de Asturias, IPLA-CSIC, Paseo Rio Linares s/n, Asturias, Spain

## Abstract

We here report a 2,801,031-bp annotated draft assembly for the *Lactococcus lactis* subsp. *cremoris* GE2-14 genome. This dairy strain produces the biogenic amine putrescine. This sequence may help identify the mechanisms regulating putrescine biosynthesis and throw light on ways to reduce its presence in fermented foods.

## GENOME ANNOUNCEMENT

*Lactococcus lactis* is of great economic importance, providing starter cultures for the production of fermented dairy products (1). However, some strains of *L. lactis* can produce putrescine, a toxic biogenic amine (BA) (2, 3).

BA are formed and accumulated in food via microbial metabolism (2). Their ingestion can cause intoxication symptoms (4), and thus, there is a consensus regarding the reduction of BA in foods (5). In dairy products, BA can reach high concentrations (6), with putrescine one of the most abundant (7). Putrescine is mainly produced by the deimination of agmatine (an arginine decarboxylation product) via the agmatine deiminase (AGDI) pathway (7, 8). Environmental and technological factors affect its accumulation (2).

We here report the draft genome of *L. lactis* subsp. *cremoris* CECT 8666 (formerly GE2-14), a strain isolated from artisanal cheese (9) that produces large amounts of putrescine (3). This strain is currently used as a model strain for studying putrescine biosynthesis regulation (10).

A 0.5-kbp genomic library was constructed and subjected to 90-bp paired-end sequencing (providing 30-fold coverage) using a HiSeq 1000 System sequencer (Illumina) (performed at the Beijing Genomics Institute, China). Quality-filtered reads were assembled using Velvet software (http://www.ebi.ac.uk/~zerbino/velvet/), resulting in 243 contigs ranging from 202 to 239,606 bp. The total sequence length was 2,801,031 bp, with a G+C content of 35.4%. Annotation was performed by Era7 Bioinformatics (Granada, Spain) using the BG7 pipeline (11), and improved using BLAST analysis results (http://blast.ncbi.nlm.nih.gov). The genome contained 2,765 predicted coding sequences. Predicted copies of the 16S, 23S, and 5S rRNA genes were found, as were 53 genes for tRNAs.

Genome analysis confirmed the presence/absence of characteristics identified phenotypically in this strain (9) as carbohydrate utilization genes. Interestingly, this strain is unable to ferment arabinose, yet it possesses the genes required. Detailed analysis detected a frameshift in a permease-encoding (U725_00413) and an α-*N*-arabinofuranosidase-encoding genes (U725_00414), probably impairing l-arabinose utilization. The genome contained at least four prophages. Indeed, almost one-tenth of the genes (285) were phage related. Several genes with homology to plasmid replication and mobilization proteins were also found, consistent with the presence of at least four plasmids.

The AGDI cluster involved in putrescine production comprised five genes, *aguR*, *aguB*, *aguD*, *aguA*, and *aguC* (U725_01346 to U725_01350). The AGDI cluster is known to be subject to carbon catabolite repression mediated by the catabolite control protein CcpA (10). Interestingly, in addition to the *ccpA* gene (U725_00364), a second gene involved in carbon catabolite repression (*ccpB*; U725_02733) was identified, although its role in AGDI cluster expression is unknown.

The availability of the *L. lactis* subsp. *cremoris* CECT 8666 genome sequence opens up the possibility of performing transcriptional studies for identifying the genes involved in the regulation of putrescine production. This would improve our knowledge of the factors affecting BA production and accumulation in dairy products, leading to improvements in food safety.

### Nucleotide sequence accession numbers.

The results of this whole-genome shotgun project were deposited in the DDBJ/EMBL/GenBank database under accession number AZSI00000000 (BioProject PRJNA225671). The version of the genome described here is version AZSI01000000.
